# Retrospective Review of Positive Newborn Screening Results for Isovaleric Acidemia and Development of a Strategy to Improve the Efficacy of Newborn Screening in the UK

**DOI:** 10.3390/ijns10010024

**Published:** 2024-03-13

**Authors:** Rachel S. Carling, Katy Hedgethorne, Anupam Chakrapani, Patricia L. Hall, Nick Flynn, Toby Greenfield, Stuart J. Moat, Joshua Ssali, Lynette Shakespeare, Nazia Taj, Teresa H. Y. Wu, Mark Anderson, Arunabha Ghosh, Hugh Lemonde, Germaine Pierre, Mark Sharrard, Sreevidya Sreekantam, James R. Bonham

**Affiliations:** 1 Biochemical Sciences, Synnovis, Guys & St Thomas’ NHSFT, London SE1 7EH, UK; 2 GKT School of Medical Education, Kings College London, London WC2R 2LS, UK; 3 Department of Metabolic Medicine, Great Ormond Street Hospital NHSFT, London WC1N 3JH, UK; 4 Department of Laboratory Medicine and Pathology, Mayo Clinic, Rochester, MN 55905, USA; 5 Biochemical Genetics Unit, Cambridge University Hospitals NHSFT, Cambridge CB2 0QQ, UK; 6 Portsmouth Hospitals Trust, Portsmouth PO6 3LY, UK; 7 Department of Medical Biochemistry, Immunology & Toxicology, University Hospital Wales, Cardiff CF14 4XW, UK; 8 School of Medicine, Cardiff University, Cardiff CF14 4XN, UK; 9 South West Thames Newborn Screening, Epsom & St Helier Hospitals, Carshalton SM5 1AA, UK; 10 Clinical Chemistry, Sheffield Children’s NHSFT, Sheffield S10 2TH, UK; 11 Department of Clinical Biochemistry, Oxford University Hospitals NHSFT, Oxford OX3 9DU, UK; 12 Willink Biochemical Genetics Laboratory, Genomic Medicine, Manchester University NHSFT, Manchester M13 9WL, UK; 13 Great North Children’s Hospital, Newcastle Upon Tyne Hospitals NHSFT, Newcastle NE1 4LP, UK; 14 Willink Biochemical Genetics Unit, Manchester University NHSFT, Manchester M13 9WL, UK; 15 Department of Paediatric Metabolic Medicine, Evelina London Children’s Hospital, Guys & St Thomas’ NHSFT, London SE1 7EH, UK; hugh.lemonde@gstt.nhs.uk; 16 University Hospitals Bristol and Weston NHSFT, Bristol BS1 3NU, UK; germaine.pierre@uhbw.nhs.uk; 17 Paediatric Medicine, Sheffield Children’s NHSFT, Sheffield S10 2TH, UK; 18 Birmingham Women’s and Children’s Hospital, Birmingham B4 6NH, UK

**Keywords:** isovaleric acidemia, false positive, newborn screening, inherited metabolic disease

## Abstract

Since the UK commenced newborn screening for isovaleric acidemia in 2015, changes in prescribing have increased the incidence of false positive (FP) results due to pivaloylcarnitine. A review of screening results between 2015 and 2022 identified 24 true positive (TP) and 84 FP cases, with pivalate interference confirmed in 76/84. Initial C5 carnitine (C5C) did not discriminate between FP and TP with median (range) C5C of 2.9 (2.0–9.6) and 4.0 (1.8–>70) µmol/L, respectively, and neither did Precision Newborn Screening via Collaborative Laboratory Integrated Reports (CLIR), which identified only 1/47 FP cases. However, among the TP cases, disease severity showed a correlation with initial C5C in ‘asymptomatic’ individuals (*n* = 17), demonstrating a median (range) C5C of 3.0 (1.8–7.1) whilst ‘clinically affected’ patients (*n* = 7), showed a median (range) C5C of 13.9 (7.7–70) µmol/L. These findings allowed the introduction of dual cut-off values into the screening algorithm to reduce the incidence of FPs, with initial C5C results ≥ 5 µmol/L triggering urgent referral, and those >2.0 and <5.0 µmol/L prompting second-tier C5-isobar testing. This will avoid delayed referral in babies at particular risk whilst reducing the FP rate for the remainder.

## 1. Introduction

England and Wales started screening for isovaleric acidemia (IVA) in 2015, with Scotland and Northern Ireland following suit in 2017 and 2020, respectively. The screening algorithm is based on the analysis of isovalerylcarnitine (C5i) by flow injection analysis tandem mass spectrometry (FIA-MS/MS) on a dried blood spot specimen collected on day five of life. The initial identification of a condition suspected IVA result is based on a single defined cut-off-value (COV) for C5i. C5i has two common biological isobars, 2-methylbutyrylcarnitine and pivaloylcarnitine (C5p), which cannot be distinguished by FIA-MS/MS. C5p can be present in blood due to maternal use of pivalic ester pro-drugs, e.g., pivmecillinam, or pivalic acid derivatives used as emollients in some creams, including nipple balms used by breastfeeding mothers. As such, the occurrence of false positive (FP) results due to interference from C5p is well documented [1,2,3]. However, when the UK first started screening for IVA, the use of pivmecillinam was thought to be uncommon; hence, FP results were not expected to be an issue. It quickly became evident that this was not the case [4], and further investigation found that the number of prescriptions for pivmecillinam issued by General Practitioners in England had increased five-fold between July 2012 and July 2016 [5]. This coincides with Public Health England recommending the drug as an alternative therapy when there is widespread bacterial resistance to ampicillin, amoxicillin, and trimethoprim [4]. Geographical variation in prescribing patterns is evident, with higher usage presumably correlating with areas of increased antimicrobial resistance [5]. The British National Formulary currently states that pivmecillinam is contra-indicated in carnitine deficiency and that ‘FP NBS results for IVA may occur in neonates born to mothers receiving pivmecillinam during late pregnancy’. At present, pivmecillinam is not approved for use in the United States.

The aim of this study was to determine whether the FP rate for IVA could be reduced by using alternative COVs, second-tier testing, or Precision Newborn Screening via Collaborative Laboratory Integrated Reports (CLIR). For the latter, the performance and outcomes of the UK’s current IVA screening algorithm, which uses a single defined COV of 2.0 µmol/L, were compared with CLIR post-analytical clinical decision support software v2.27.

## 2. Materials and Methods

An eight-year, retrospective review of condition suspected results for IVA was performed. Information was obtained on all babies referred via the UK newborn screening program between January 2015 and December 2022, with an initial condition suspected result for IVA: date of specimen, NHS number, laboratory identifier, initial C5C result, C5 isobars result, mutation analysis, outcome, additional information relating to antibiotic use. Data were analysed using GraphPad Prism v10.0.2.

A short questionnaire designed to obtain additional information on clinical outcomes was distributed to each of the Newborn Screening Clinical Services. Clinicians were asked to classify each case as ‘asymptomatic’ or ‘clinically affected’. Each case was also classified as severe (classical IVA) or attenuated phenotype (mild IVA) using criteria described previously [6]. A copy of the questionnaire is provided in Supplementary Material S1.

A retrospective evaluation of Precision Newborn Screening via CLIR was undertaken. Reference case data, true positive (TP), and FP case data were submitted to CLIR to provide initial location-specific data. Case data included the following: age at time of specimen collection, birth weight, gestational age, sex, analyte concentration (methionine, total leucine, phenylalanine, tyrosine, C5C, octanoylcarnitine, decanoylcarnitine, glutarylcarnitine, thyroid stimulating hormone, and immunoreactivetrypsin). Post-analytical tools were customized for the UK location (GBR) to only include analytes in the UK screening panel. The shared IVA tool utilizes 22 acylcarnitines and amino acids, while the site-specific GBR tool uses only eight, reflecting the smaller analytical panel utilized [7]. A single condition tool (SCT) was created. Quantification of the utility of CLIR in correctly identifying FP IVA cases was determined by submission of additional FP cases to CLIR and subsequent analysis with the SCT and a dual scatter plot (DSP) designed to discriminate FP results from confirmed cases of IVA. Permission was obtained from the Antenatal and Newborn Screening Research and Innovation Development Advisory Committee for the retrospective evaluation of CLIR. All case data were anonymized prior to submission.

## 3. Results

### 3.1. Retrospective Review of Condition Suspected IVA Results

Between January 2015 and December 2022, 109 babies with condition suspected results for IVA were identified. Of these, 24 were TP cases, and 84 were FP cases. One ‘other condition suspected’ case was removed from subsequent data analysis. The mean (median, range) C5C result for the TP cases was 10.9 µmol/L (4.0, 1.8–>70). Of the TP cases, seven were c.941C>T homozygous, six were compound heterozygous, two of which were c.941C>T compound heterozygous, and seven cases were homozygous other. The remaining four were c.941C>T heterozygous and were classified as TP cases on the basis of increased urinary isovalerylglycine.

**Figure 1:**
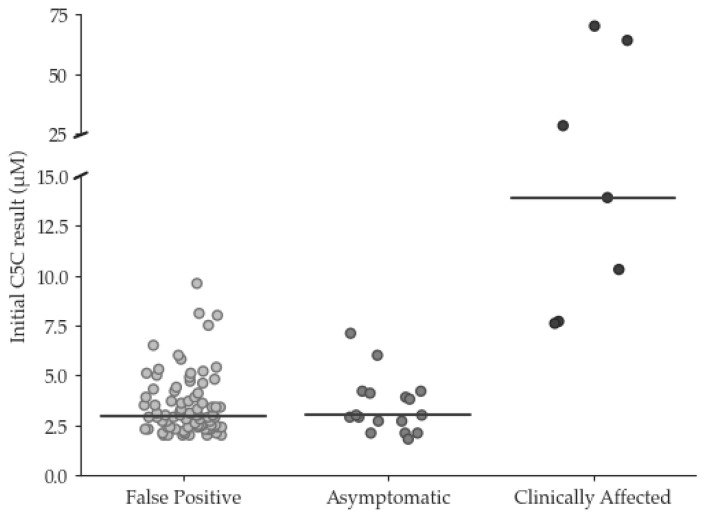
Initial C5 carnitine concentration (µmol/L) in false positive and true positive cases (median concentration denoted by horizontal bar). True positive cases are categorized as ‘asymoptomatic’ and ‘clinically affected’.

The mean (median, range) C5C result for the FP cases was 3.5 µmol/L (2.9, 2.0–9.6). The incidence of FP cases was approximately 0.0015%. The initial C5C results for the TP and FP cases are summarised in Figure 1. Pivalate interference was confirmed in 67/84 FP cases by C5 isobar analysis [4]. Although 17/84 cases did not have isobar analysis performed, 9/17 had documented evidence of maternal pivampicillin use. The remaining eight cases had unremarkable urine organic acid and bloodspot acylcarnitine profiles.

**Figure 2:**
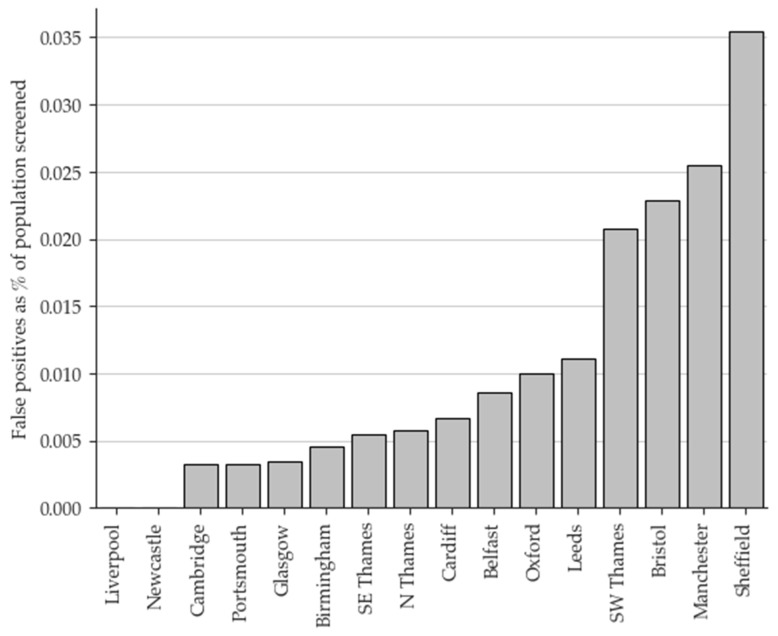
False positive results by screening laboratory (2015–2022) shown as a percentage of the population screened. North Thames, South East Thames, and South West Thames laboratories are all based in London.

The overall FP rate for the eight-year period was 78%. Geographical variation in the FP rate was evident, as illustrated in Figure 2. Of all the FP results, 26.7% occurred in just one laboratory, and 55.8% occurred in just three. Conversely, two laboratories have not had an FP case to date.

### 3.2. Clinical Outcome Questionnaire

The clinical outcome questionnaire was sent to the Lead Paediatric Metabolic Consultant at the following hospitals that run the Newborn Screening Clinical Services: Birmingham, Bristol, Evelina London, Great Ormond Street, Manchester, and Sheffield Children’s Hospitals. Responses were received from 6/6 centres. Of the 24 TP cases, 7 were classified as ‘clinically affected’, and 17 were classed as ‘asymptomatic’ (see Figure 1). Of the ‘clinically affected’ children, 5/7 experienced further episodes of decompensation since diagnosis, and all were being treated with a combination of a protein-restricted diet and emergency regimen (ER). Moreover, 2/7 were also receiving glycine supplementation, 2/7 were receiving carnitine supplementation, and 3/7 were being supplemented with both. Of the ‘asymptomatic’ children, 15 were being managed on an ER only, 2/15 were receiving an ER with mild protein restriction and carnitine supplementation, and 1/2 was also receiving glycine supplementation.

### 3.3. Evaluation of Precision Newborn Screening

**Figure 3:**
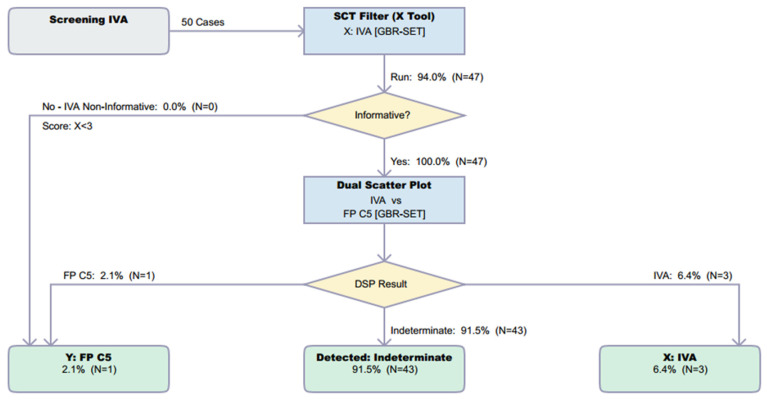
Resolution by CLIR Dual Scatter Plot Runner of 50 FP cases with increased C5C result. Image is shown unedited, as created automatically by the software. Colour coding is as follows: Grey, start; Blue, process; Yellow, decision; Green, totals.

Preliminary data submitted to CLIR included *n* = 288,735 reference cases, *n* = 4 TP IVA cases, and *n* = 34 FP cases due to C5p interference. An additional *n* = 50 FP cases were subsequently submitted to CLIR and analysed using the DSP (see Figure 3). Additionally, 3/50 cases were rejected due to missing decanoylcarnitine results. Of the 47 cases remaining, 1/47 was correctly identified as FP, 3/47 were incorrectly classified as TP IVA, and 43/47 were classified as ‘indeterminate’. In a screening environment, indeterminate results typically require follow-up, although programs may choose to use this as a group that requires a repeat specimen rather than a referral for confirmatory testing. It may also serve as a useful categorization tool for second-tier testing [7,8].

## 4. Discussion

A widely cited quotation draws attention to both the harms and benefits of screening—‘All screening programmes do harm; some do good as well, and, of these, some do more good than harm at reasonable cost’ [9].

The potential harms resulting from newborn screening include both the generation of FP results, with its psychosocial impact on families, and uncertainty in TP cases resulting from a diverse clinical phenotype, making decisions about risk and treatment difficult both for physicians and parents. Furthermore, FP results also have a financial impact, with families making trips to specialist clinical centres, which are often not geographically close by. The results from this retrospective study of screening for IVA over eight years in the UK illustrate the practical implications of such disbenefits and the need to continually review and improve current screening practices.

Whilst the geographical variation in FP results has been linked to the prescribing patterns of pivmecillinam, it is important to consider whether inter-laboratory variation is also a factor. Whilst all UK screening laboratories adhere to a defined algorithm with common COVs, equipment and methodology differ and inter-instrument variation of C5 has previously been reported to be 46% [10]. However, in addition to standard proficiency testing, population data has been monitored centrally on a quarterly basis since 2015 and confirms that the variation is not related to inter-laboratory bias (See Supplementary Material Figure S1).

The findings from 108 screen-positive cases indicate that 84 were FP (78%), while among the 24 TP cases identified, only 7 required classical treatment with protein restriction and supplementary glycine and/or carnitine, with 17/24 true positive cases remaining asymptomatic with less onerous interventions.

These results emphasize the responsibility of those conducting screening to continually review and improve the specificity of newborn screening and, where possible, more closely define the prognosis in true positive cases.

**Figure 4:**
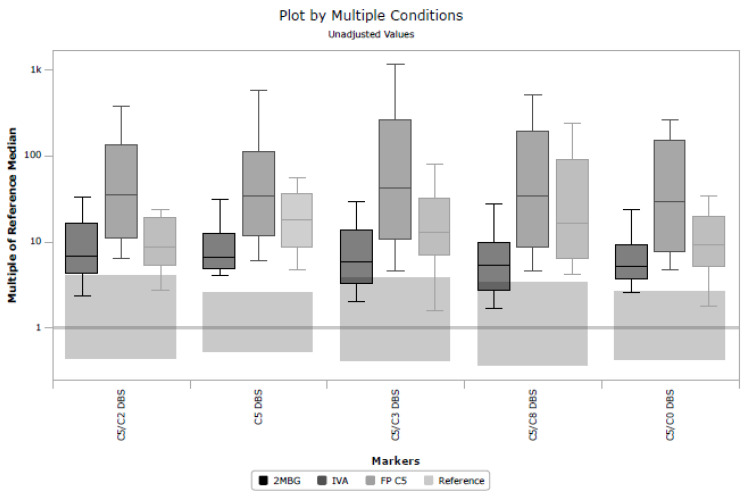
Plot by multiple conditions illustrating the similarities between isovaleric acidemia, 2-methylbutyrylglycinuria and false positive C5 cases for five key markers; C5/C2; C5; C5/C3 ratio; C5/C8 ratio and C5/C0 ratio.

Unfortunately, in a UK context, post-analytical tools in CLIR provided limited utility in eliminating samples known to be FP. There are multiple factors causing this performance, but a key driver to CLIR’s success is taking advantage of multiple analytes and combining small features to discriminate between very similar profiles, for example, 2-methylbutyrylglycinuria, isovaleric acidemia, and FP C5 cases. Figure 4 shows a plot of multiple conditions based on the cumulative data in CLIR and illustrates the similarities between these three conditions. The largest elevations in C5 are associated with TP IVA, but milder elevations can be seen in all three conditions. That utility was not able to be fully exploited in this study due to the limited panel of analytes included in the UK. The shared single-condition tool for IVA in CLIR utilizes 21 analytes (a combination of amino acids and acylcarnitines); however, for the location-specific tool created for this study, only eight analytes were available. Furthermore, age at the time of specimen collection was only available in whole days, whereas CLIR was designed to be used with age in hours. Additional case data and the further stratification of TP and FP cases, for example, ‘IVA symptomatic’, ‘IVA asymptomatic’, and FP IVA, may allow for the creation of more specifically targeted tools. Post-analytical tools in CLIR can be customized for a specific location; thus, a rule to mimic the higher symptomatic IVA cutoff could be included if a specific location were aggressively targeting FP reduction.

An alternative solution to help reduce false positive results due to C5p would be to introduce C5 isobar analysis as a second-tier screening test [4,11,12,13,14]. This would successfully address the problem of false positives due to pivalate and prevent the unnecessary referral of these babies. Second-tier testing protocols are becoming increasingly common in screening programs around the world and enabled additional disorders, e.g., disorders of propionate metabolism, classical homocystinuria and remethylation disorders, maple syrup urine disease, guanidinoacetate methyl transferase deficiency, to be included in existing programs whilst minimizing FP rates and improving the efficacy of screening [15,16,17,18,19]. There are, however, practical issues to consider in this context, as screen-positive results for IVA are relatively rare. In the UK, there are 16 newborn screening laboratories, and the evidence suggests approximately 14 screen-positive cases for IVA per year; this indicates that a typical laboratory would be required to undertake isobar analysis only once per year. Ensuring the quality and robustness of the C5 isobar test, maintaining accreditation, and being able to deliver the test when required, at short notice, poses a challenge for several laboratories.

A more immediate solution to mitigate the challenge associated with all laboratories providing second-tier testing may be to centralise C5 isobar testing at a small number of centres, although at least two laboratories would be required. This would help maintain a robust service and facilitate continuous competency, external quality assessment, and accreditation requirements. However, a drawback of this approach is the resulting delay in clinical referral whilst specimens are transported between laboratories for testing. Fortunately, the initial screening result appears to correlate with disease severity and, consequently, risk; it is notable that all 17 TP cases with initial C5C concentration < 7.2 µmol/L remained asymptomatic, whilst those requiring more intensive care demonstrated initial C5C results > 7.5 µmol/L. This is broadly comparable with the finding from the German NBS programme, where a review of 84 individuals with IVA confirmed by NBS concluded that an initial C5C concentration < 5.6 µmol/L was associated with asymptomatic disease course in most cases [20].

These findings indicate that isobar analysis could be safely conducted in those babies in whom the initial C5C result was less than a conservative COV of 5.0 µmol/L, even though this would introduce a short delay (three to four days) in clinical referral while maintaining immediate referral for those babies whose initial C5C results were over 5.0 µmol/L and likely to be at greater risk. These data suggest that this tiered approach to secondary testing would significantly reduce the burden of FP results when screening for IVA while avoiding the risk of delay for babies whose screening test results indicate that immediate intervention may be warranted.

## Data Availability

The data that support the findings of this study are available upon request from the corresponding author. The data are not publicly available due to privacy or ethical restrictions.

## Supplementary Materials

The following supporting information can be downloaded at: https://www.mdpi.com/article/10.3390/ijns10010024/s1, Supplementary Material S1: Example of questionnaire, Figure S1: Example of population centiles for C5 carnitine by UK laboratory for quarter 3htree, 2021. Where laboratories have two instruments, this is denoted by 1 and 2. Violin plot shows 25th and 75th centiles with median shown by the line, whiskers show 10th and 90th centile.
