# Supplementary material for: Retrospective Review of Positive Newborn Screening Results for Isovaleric Acidemia and Development of a Strategy to Improve the Efficacy of Newborn Screening in the UK
Source: Int J Neonatal Screen. 2024 Mar 13;10(1):24. doi: 10.3390/ijns10010024 (PMC10970767; doi:10.3390/ijns10010024)
Supplement: Supplementary file 1 [file IJNS-10-00024-s001.zip › IJNS-2861948-supplementary.pdf]

S1: Retrospectively review of true positive newborn screening results for isovaleric acidemia (January 2015 to December 2022).

Clinical outcome questionnaire (please complete a separate template for each child)

NHS Number:

Date of screening referral:

Is the child clinically affected or is the child asymptomatic?

If symptomatic, was the initial presentation prior to the screening result becoming available and on what day of life?

What was the initial presentation (clinical and lab)?

Has the child any had further episodes of decompensation?

Any other symptoms of IVA on follow up?

Homo/heterozygous for 941C>T mutation?

Was there a family history prior to screening?

Is the child on treatment?

Diet?

Supplementary Material S1: Example of questionnaire

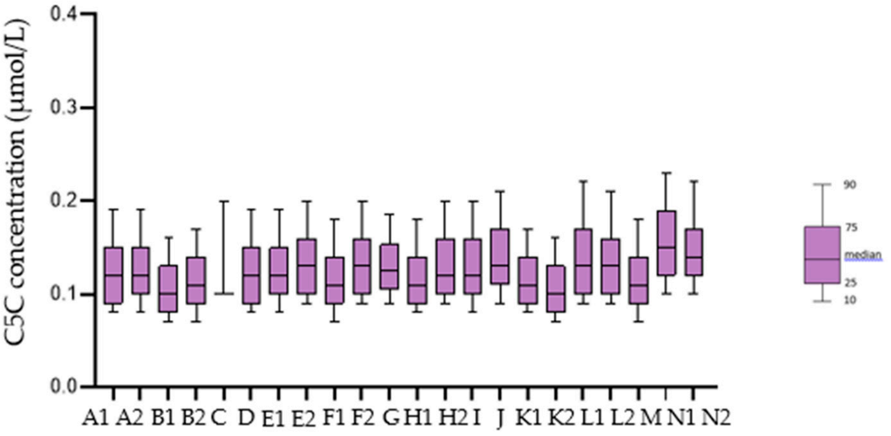

Figure S1: Example of population centiles for C5 carnitine by UK laboratory for quarter three, 2021. Where laboratories have two instruments, this is denoted by 1 and 2. Violin plot shows 25th and 75th centiles with median shown by the line, whiskers show 10th and 90th centile.
